# Structural analysis of disease-related TDP-43 D169G mutation: linking enhanced stability and caspase cleavage efficiency to protein accumulation

**DOI:** 10.1038/srep21581

**Published:** 2016-02-17

**Authors:** Chien-Hao Chiang, Cédric Grauffel, Lien-Szu Wu, Pan-Hsien Kuo, Lyudmila G. Doudeva, Carmay Lim, Che-Kun James Shen, Hanna S. Yuan

**Affiliations:** 1 Institute of Molecular Biology, Academia Sinica, Taipei, 11529, Taiwan; 2 Institute of Bioinformatics and Structural Biology, National Tsing Hua University, Hsin Chu, 30013, Taiwan; 3 Institute of Biomedical Sciences, Academia Sinica, Taipei, 11529, Taiwan

## Abstract

The RNA-binding protein TDP-43 forms intracellular inclusions in amyotrophic lateral sclerosis (ALS). While TDP-43 mutations have been identified in ALS patients, how these mutations are linked to ALS remains unclear. Here we examined the biophysical properties of six ALS-linked TDP-43 mutants and found that one of the mutants, D169G, had higher thermal stability than wild-type TDP-43 and that it was cleaved by caspase 3 more efficiently, producing increased levels of the C-terminal 35 kD fragments (TDP-35) *in vitro* and in neuroblastoma cells. The crystal structure of the TDP-43 RRM1 domain containing the D169G mutation in complex with DNA along with molecular dynamics simulations reveal that the D169G mutation induces a local conformational change in a β turn and increases the hydrophobic interactions in the RRM1 core, thus enhancing the thermal stability of the RRM1 domain. Our results provide the first crystal structure of TDP-43 containing a disease-linked D169G mutation and a disease-related mechanism showing that D169G mutant is more susceptible to proteolytic cleavage by caspase 3 into the pathogenic C-terminal 35-kD fragments due to its increased stability in the RRM1 domain. Modulation of TDP-43 stability and caspase cleavage efficiency could present an avenue for prevention and treatment of TDP-43-linked neurodegeneration.

Protein misfolding can lead to the formation of intra or extracellular accumulation of aggregated protein and is frequently associated with various pathological conditions such as neurodegeneration. One example is TDP-43, which binds RNA and DNA and functions in mRNA splicing, translation regulation and transport^1^. However, when misfolded, TDP-43 aggregates and forms the major component in the ubiquitylated inclusions in the neuronal cells of patients with amyotrophic lateral sclerosis (ALS) and frontotemporal lobar degeneration (FTLD)^2^,^3^. TDP-43 forms cytoplasmic ubiquitylated inclusions in about half of the FTLD patients and all the ALS patients^1^,^4^,^5^. The disease form of TDP-43 in ALS and FTLD is not only hyperphosphorylated and ubiquitinated but also proteolytically cleaved into C-terminal fragments of about 35 and 25 kD^6^,^7^. The inclusion of TDP-43 has also been characterized in various neurodegenerative disorders, linking its aggregation to a wide spectrum of neurodegenerative pathologies^1^.

TDP-43 consists of an N-terminal domain (NTD) and two tandem RNA recognition motifs, RRM1 and RRM2, followed by a C-terminal glycine-rich region (G) (Fig. 1A). It forms homodimers and the two RNA-recognition motifs are involved in nucleic acid binding^8^,^9^,^10^,^11^. About 5% of ALS cases are familial and linked to mutations in several genes, including ~3% in *TARDBP*, the gene encoding TDP-43^12^,^13^. About fifty missense mutations in *TARDBP* have been identified in familial and sporadic ALS, most of which are located in the C-terminal G-rich region with only two exceptions to-date, A90V in the NTD and D169G in the RRM1^13^,^14^,^15^,^16^,^17^,^18^,^19^,^20^,^21^,^22^,^23^,^24^,^25^,^26^,^27^,^28^,^29^,^30^,^31^,^32^,^33^,^34^,^35^,^36^,^37^. Although the crystal structures of the TDP-43 RRM1 and RRM2 bound to a single-stranded DNA^8^,^9^ as well as the NMR solution structure of RRM1-RRM2 bound to a single-stranded RNA^38^ have revealed how the two RNA recognition motifs interact with nucleic acids, they do not provide a clear link between missense mutations in TDP-43 and ALS.

ALS-linked mutants have been suggested to be more prone to aggregation and more potent in inducing cell death than wild-type TDP-43: Compared to the latter, Q331K and M337V accelerate spontaneous TDP-43 aggregation^39^, A315T, G348C and A382T induce neuron cell death^40^,^41^, and Q331K and M337V more strongly induce oxidative injury of motor-neuron like cells^42^. Moreover, the ALS-linked mutants, G298S, Q331K, M337V, exhibit longer protein half-lives and enhanced interactions with FUS/TLS^43^, whereas the D169G mutation in the RRM1 or RRM1-RRM2 domain increased protein thermal stability^9^ or half-life^44^. The D169G TDP-43 mutant reduces the binding affinity to ubiquilin 1 (UBQLN) and oxidative resistance 1 (Oxr1)^45^,^46^. Interestingly, early ALS disease onset has been correlated with the increased stability of TDP-43 from 12.6 hours for the wild-type to 15.9–50.6 hours for seven ALS-linked mutants, G298S, A315T, M337V, Q343R, G348C, N352S, A382T, suggesting that TDP-43 protein stability is directly associated with ALS pathogenic pathways^47^.

However, it is still unknown why some ALS-linked mutants exhibit increased stability with longer half-lives and how these properties contribute to protein accumulation and disease onset. Progranulin-mediated cleavage of TDP-43 by caspases 3 and 7 may generate the 25 kD (TDP-25) and 35 kD (TDP-35) C-terminal fragments^48^ (see Fig. 1A). The proteolytic cleavage of TDP-43 by caspases is suggested to be the major intermediate step for the clearance of TDP-43^49^,^50^. Therefore blockage of caspase digestion to reduce TDP-25 and TDP-35 levels results in severe delay in TDP-43 clearance and prolonged cell death^50^,^51^,^52^,^53^. In contrast, overexpression and high levels of TDP-25 and TDP-35 in cultured cells enhance the formation of insoluble TDP-43 aggregates^49^,^54^,^55^,^56^,^57^. The roles of TDP-25 and TDP-35 in TDP-43 aggregation and clearance remain enigmatic.

Here, we provide biochemical, structural, simulation, and cellular data to show that compared to wild-type TDP-43, the ALS-linked D169G mutation in TDP-43 generates a more stable protein that is cleaved by caspase 3 more efficiently, producing increased levels of TDP-35, but not TDP-25. The crystal structure of the RRM1 domain containing the D169G mutation, along with molecular dynamics simulations, provides a molecular basis as to how this mutation increases protein stability. We suggest that TDP-35 bearing the RRM1 domain is stabilized by the D169G mutation and is thus the favored product in caspase digestion of TDP-43. This result provides the first crystal structure of TDP-43 containing a disease-associated mutation and the working mechanism showing that the D169G mutation renders TDP-43 more susceptible to production of the pathogenic C-terminal fragment TDP-35, thus linking it to ALS.

## Results

### TDP-43 mutant D169G exhibits increased thermal stability

**Figure 1:**
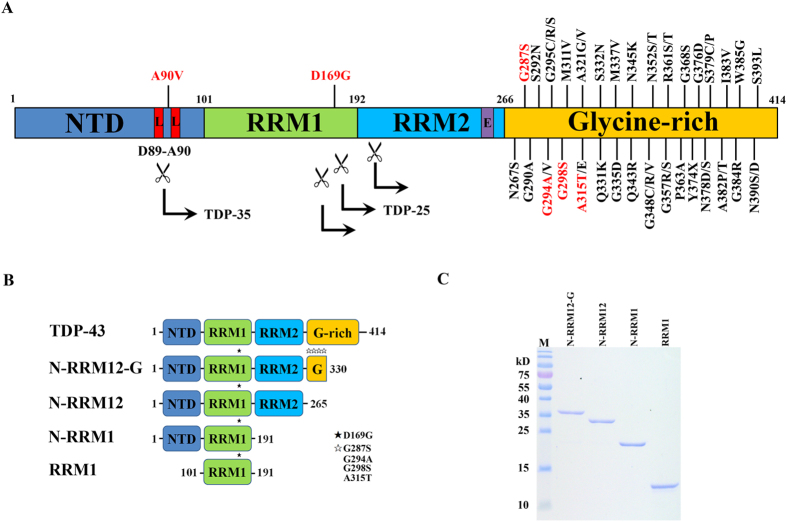
TDP-43 domain structure and the ALS-linked mutations. (**A**) TDP-43 consists of an N-terminal domain (NTD), two tandem RNA recognition motifs (RRM1 and RRM2), and a C-terminal glycine-rich region. About fifty missense mutations have been identified in TDP-43 in the sporadic and familial ALS patients, mostly localized in the glycine-rich region. Six mutations studied here are displayed in red. The scissile indicates the caspase cleavage sites that generate the C-terminal fragments of TDP-35 (the C-terminal 35 kD fragments) and TDP-25 (the C-terminal 25 kD fragments). L stands for nuclear localization signal (NLS) and E stands for nuclear export signal (NES). (**B**) Different truncated species of TDP-43 were constructed, N-RRM12-G (residues 1 to 330), N-RRM12 (residues 1 to 265), N-RRM1 (residues 1 to 191) and RRM1 (residues 101–191). The black and white stars indicate the mutations studied here. (**C**) The purity of the truncated TDP-43 proteins shown by the SDS-PAGE.

To examine the effects of the TDP-43 mutations on protein stability, we first expressed the full-length His-tagged human TDP-43 in *E. coli*. However, the recombinant full-length TDP-43 was highly unstable and degraded within hours of its expression, suggesting that the C-terminal G-rich tail destabilizes TDP-43. We further expressed the His-tagged truncated TDP-43 with a shorter C-terminal G-rich tail and identified a more stable truncated form of TDP-43 (residues 1–330), which could be analyzed in biochemical assays within 24 hours after its expression and purification. This TDP-43 (residues 1–330) is referred to as N-RRM12-G, as it had complete NTD, RRM1 and RRM2 domains, and a C-terminal glycine-rich tail of about 65 residues (see Fig. 1B). For a comparison in biochemical properties, we also expressed and purified various truncated forms of TPD-43, including N-RRM12 (residues 1–265), N-RRM1 (residues 1–191) and RRM1 (residues 101–191) (Fig. 1B,C) and measured their thermal stabilities by monitoring the melting of the β-strand structure at 218 nm using circular dichroism.

**Figure 2:**
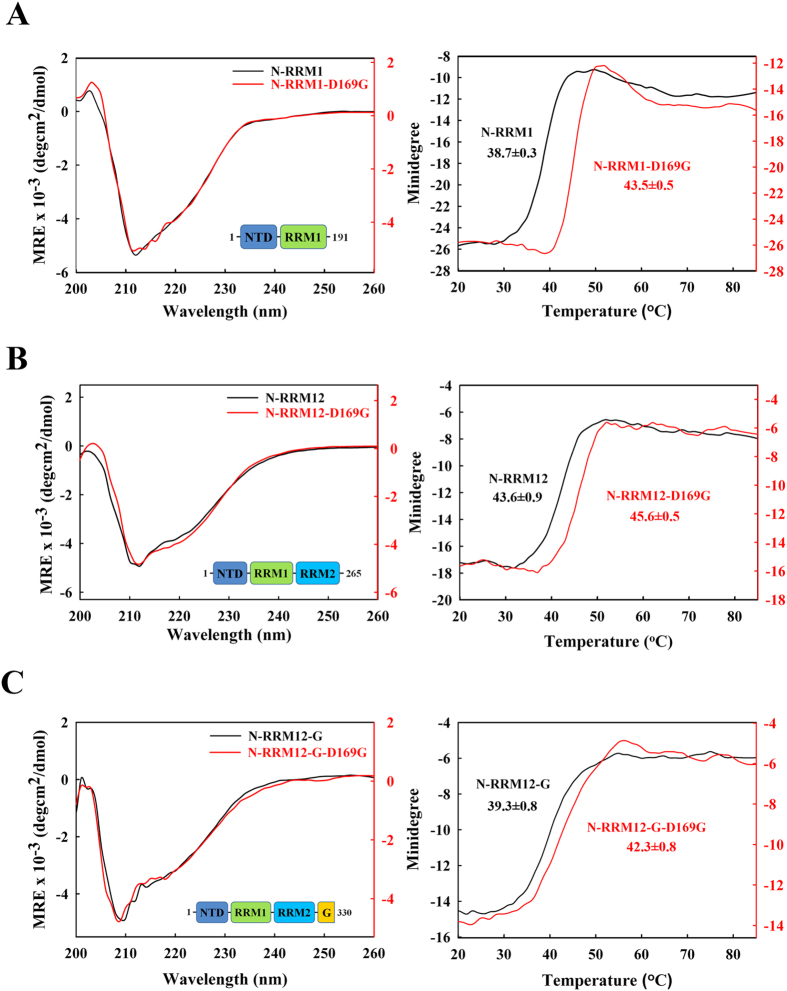
TDP-43 D169G mutants are more resistant to thermal denaturation as monitored by circular dichroism. The CD spectra were scanned from 260 to 200 nm for three times at 25 °C for different truncated constructs of TDP-43: (**A**) N-RRM1 and D169G mutant; (**B**) N-RRM12 and D169G mutant; (**C**) N-RRM12-G and D169G mutant. Melting temperatures were measured at the loss of the β-strand structure at 218 nm for wild-type TDP-43 and D169G mutant. The melting points (Tm) are labeled in each melting curve.

**Figure 3:**
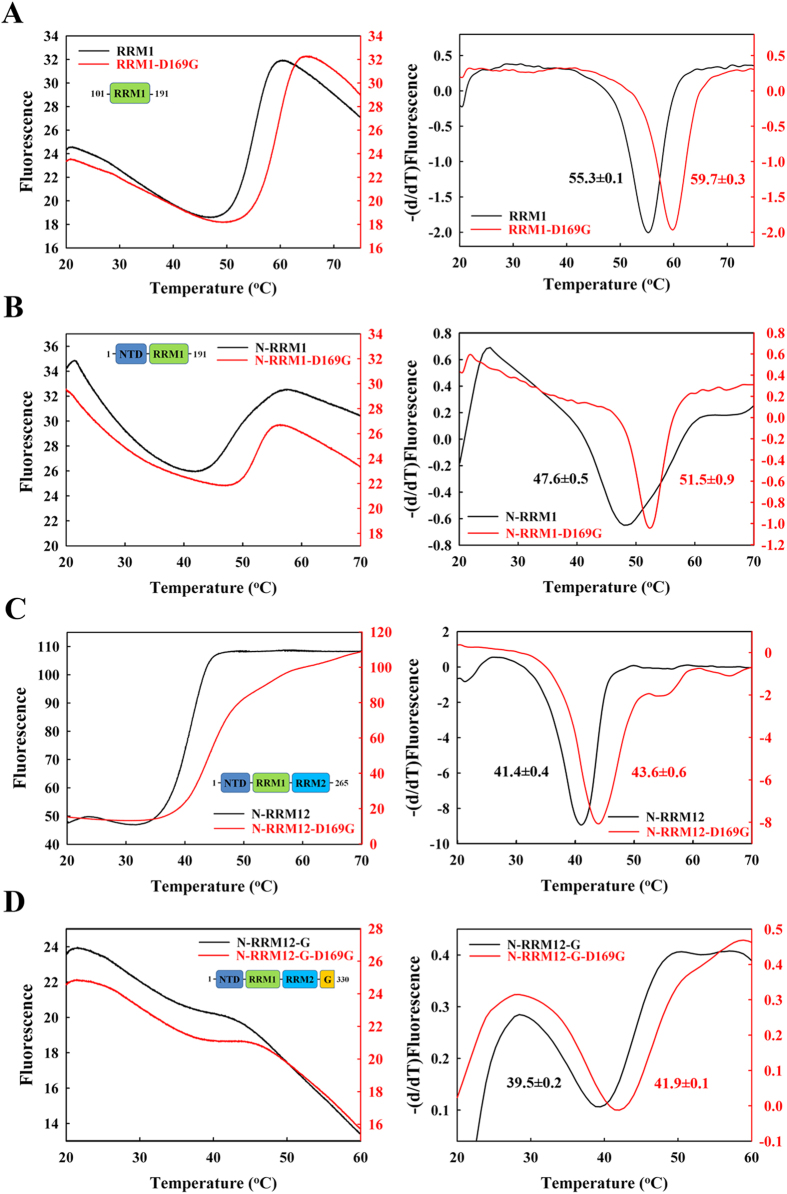
TDP-43 D169G mutants are more resistant to thermal unfolding as monitored by differential scanning fluorimetry. The overall folding stability of TDP-43 was assayed by differential scanning fluorimetry for (**A**) RRM1 and D169G mutant; (**B**) N-RRM1 and D169G mutant; (**C**) N-RRM12 and D169G mutant; (**D**) N-RRM12-G and D169G mutant. The temperature was increased from 20 to 85 °C at a heating rate of 0.012 °C/second, and the emitted fluorescence at 568 nm was recorded every 0.02 °C.

The results in Fig. 2A–C and Table 1 show that the smallest construct, RRM1, was the most thermally stable with a melting point of 53 ± 0.9 °C, whereas the NTD reduced its stability (with a much lower melting point of 38.7 ± 0.3 °C for N-RRM1), and the C-terminal tail decreased the stability of TDP-43 (lower melting point of 39.3 ± 0.8 °C for N-RRM12-G compared to that of 43.6 ± 0.9 °C for N-RRM12). To confirm these results, the stabilities of the various TDP-43 constructs were further assayed by differential scanning fluorimetry at 568 nm (Fig. 3 and Table 1). Consistent with CD, N-RRM12-G had a lower melting point of 39.5 ± 0.2 °C compared to N-RRM12 (41.4 ± 0.4 °C). In summary, these results suggest that adding the NTD and the C-terminal glycine-rich tail to the RRM domains decreases the stability of TDP-43.

We then expressed and purified mutant N-RRM12-G containing six ALS-linked mutations: A90V, D169G, G287S, G294A, G298S and A315T (red mutations in Fig. 1A). Compared to the wild-type protein, D169G had a significantly increased melting point of +3.0 °C as monitored by CD, whereas the remaining mutants had smaller differences ranging from −1.6  °C to 0.5 °C (Table 1). This result is consistent with our previous finding showing that the RRM1 domain with the D169G mutation is more thermally stable^9^ and the report showing that RRM1-RRM2 containing the D169G mutation exhibits a longer cellular half-life^44^. To further examine the effects caused by this mutation, we measured the melting points of D169G in the different constructs by CD and differential scanning fluorimetry (Figs 2 and 3). We found that the D169G mutation consistently enhanced thermal stability in all TDP-43 constructs examined, with a larger melting point increase of 7–8 °C for the smaller RRM1 and N-RRM1 proteins and a smaller increase of 2–3 °C for the larger N-RRM12 and N-RRM12-G proteins (Table 1). These results suggest that the ALS-linked mutation D169G increases the thermal stability of TDP-43 and this effect is more significant in a smaller TDP-43 construct, such as RRM1.

### D169G is more efficiently cleaved by caspase 3 to TDP-35 fragments than wild-type TDP-43

How could the enhanced stability of TDP-43 be linked to ALS? To answer this intriguing question, we treated wild-type TDP-43 (N-RRM12-G) and the corresponding D169G mutant with caspases 3, 4, 6 and 7, since it has been suggested that TDP-35 is generated mainly through caspase 3/7-mediated cleavage at D89–A90^48^, whereas various TDP-25 fragments could be generated through caspase 3 cleavage at D208-V209^48^, caspase 4 cleavage at D174–C175 or caspase 6/7 cleavage at D169–G170^50^ (Fig. 1A). However, N-RRM12-G was not stable adequately, making it difficult to evaluate the caspase-mediated cleavage difference between wild-type TDP-43 and D169G mutant (data not shown). Hence, we treated the shorter construct of wild-type TDP-43 (N-RRM12) and its two mutants within the construct (residues 1 to 265), A90V and D169G, with caspases.

**Figure 4:**
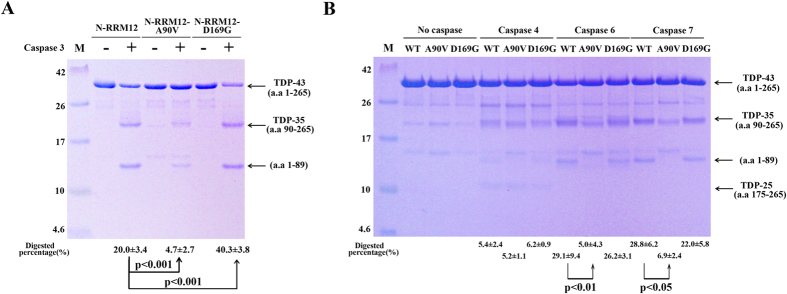
TDP-43 D169G mutant is more susceptible to proteolytic cleavage by caspase 3. (**A**) The wild-type, A90V and D169G mutant of TDP-43 (N-RRM12, residues 1–265) were incubated with caspase 3 for two hours. The SDS-PAGE stained with Coomassie blue revealed that TDP-43 was cleaved into TDP-35. Tandem mass spectrometry confirmed that caspase 3 digested N-RRM12 into two major fragments, TDP-35 (residues 90–265), and residues 1–89. (**B**) The wild-type, A90V and D169G mutant of TDP-43 (N-RRM12) were incubated with caspase 4, 6 and 7 for 16 hours. The digested proteins were resolved by 15% Tricine-SDS-PAGE with Coomassie blue staining. The mean percentages with standard errors shown at the bottom of the gel were calculated from three independent experiments. Statistical significance P values were determined by an unpaired two-tailed Student’s *t*-test.

Compared to wild-type TDP-43, the D169G mutant was cleaved by caspase 3 more efficiently yielding an increased level of TDP-35: Caspase 3 digested the D169G mutant at twice the percentage level (40.3 ± 3.8%) of the wild-type protein (20 ± 3.4%, see Fig. 4A). Tandem mass spectrometry analysis of the cleaved fragments confirmed cleavage between D89 and A90 (data not shown), yielding mainly TDP-35 (residues 90–265) and an N-terminal fragment (residues 1–89). On the other hand, the D169G mutant was cleaved by caspase 4, 6 or 7 with an efficiency similar to that of wild-type TDP-43 (see Fig. 4B). Moreover, only caspase 4 digested TDP-43 into TDP-25 (residues 175–265), whereas caspase 6/7 digested TDP-43 into primarily TDP-35. Thus, compared to wild-type TDP-43, the D169G mutant was more susceptible to caspase 3 digestion, yielding more TDP-35, but was cleaved similarly by caspase 4, 6 and 7, yielding similar levels of TDP-25 and TDP-35. The mutation of D169 did not reduce the level of TDP-25 in our *in vitro* digestion experiments (see Fig. 4B). One of the reasons is likely because the TDP-25 fragments could be generated by many cleavage sites between D208/V209, D174/C175 and D169/G170, and the cleavages were not solely dependent on D169/G170. Or it is possible that the band of TDP-25 in the gel was too faint to reveal any difference. A previous cellular study shows a similar result that transient expression of TDP-43 with D169G mutation in U2OS cells produced a little effect in the production of TDP-25 in the early stages but the clearance rate for the full-length TDP-43 was largely reduced^50^. It is likely that the cleaved TDP-35 containing the D169G mutation is more stable than the full-length TDP-43 so that it is preferably generated by caspase digestion (see Discussion).

In contrast to the D169G mutant, the A90V mutant was cleaved less efficiently (~5%) by caspase 3 than wild-type TDP-43 (~20%), likely because A90 is an important determinant for caspase 3 cleavage being right next to the cleavage site of D89–A90 (Fig. 4A). Likewise, A90V was more resistant to caspase 6 (~5%) and caspase 7 (~7%) cleavage, as compared to the wild-type protein (~29% and ~30%) (Fig. 4B). However, both wild-type and A90V TDP-43 were similarly cleaved inefficiently (~5%) by caspase 4, generating similar levels of TDP-25. In summary, compared to wild-type TDP-43, the A90V mutant was more resistant to caspase 3, 6 and 7 cleavage generating TDP-35 but was similarly cleaved by caspase 4 in generating TDP-25.

### Crystal structure of RRM1-D169G in complex with DNA reveals a local conformational change

**Figure 5:**
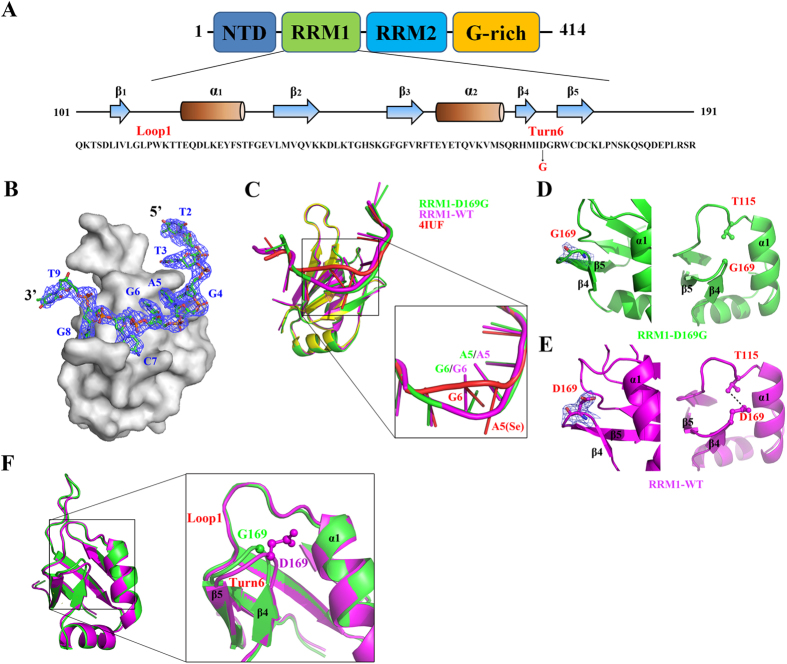
Crystal structures of RRM1/DNA and RRM1-D169G/DNA complexes. (**A**) The sequence and secondary structure of the RRM1 domain in TDP-43. (**B**) The overall crystal structure of RRM1-D169G/DNA complex demonstrates that eight nucleotides (T2 To T9) have well defined electron density as shown by the difference Fourier (2Fo-Fc) map. (**C**) The superimposition of RRM1/DNA (PDB entry code: 4IUF) determined previously with RRM1/DNA and RRM1-D169G/DNA determined in this study reveals that the conformation of the Se-modified DNA (red) deviates from the unmodified DNA (magenta and green). (**D**) The D169G mutant has almost no electron density around G169 side chain, as shown by the omit map in the left panel. No interaction was observed between G169 and T115, as shown in magnification in the right panel. (**E**) The wild-type RRM1 has well-defined side-chain electron density for D169 as shown in the omit map in the left panel. The hydrogen bond between D169 and T115 is displayed in the right panel. (**F**) The crystal structure of wild-type RRM1 (magenta) is superimposed with RRM1-D169G (green) complex. A close look at Turn6 shows that this turn shifts in the D169G mutant.

To reveal the molecular basis for the increased stability rendered by the D169G mutation, we co-crystallized RRM1 with DNA and determined the crystal structures of the wild-type RRM1 and RRM1-D169G mutant in complex with a single-stranded DNA, 5′-GTTGAGCGTT-3′ (see RRM1 sequence in Fig. 5A). This DNA was chosen as it had been used to co-crystallize with TDP-43 RRM1 and RRM2 domain^8^,^9^. Unlike the crystal structure (PDB entry: 4IUF) of RRM1 bound to the same DNA with a Se-H attached at the 2′-C of the sugar ring of adenine A5^9^, the present crystal structures of RRM1 and RRM1-D169G were bound to DNA without any modification. The RRM1/DNA and RRM1-D169G/DNA crystal structures were determined by molecular replacement using the 4IUF structure as the search model. After refinement of the RRM1 domain structure, the difference Fourier map (Fo-Fc) revealed a clear continuous electron density that could be fitted respectively with 8 and 9 nucleotides in the two RRM1/DNA complexes per asymmetric unit (Fig. 5B and Supplementary Figure 1). For the RRM1-D169G/DNA complex, the final refined model contained four RRM1 polypeptide chains (chain A to D) and four single-stranded DNA per asymmetric unit (Supplementary Figure 1). For clarity, only a single RRM1/DNA (chain A) and RRM1-D169G/DNA (chain A) were used for structural presentation and comparison hereafter. The diffraction and refinement statistics of RRM1/DNA (RCSB code 4Y0F) and RRM1-D169G/DNA (RCSB code 4Y00) are listed in Table 2. Superposition of the RRM1/DNA complexes determined in this and previous studies reveal that the unmodified DNA bound in RRM1 and RRM1-D169G (this study) fit well, but deviate from the Se-labeled DNA around the Se-labeled sites at A5 and G6 (see Fig. 5C).

The overall structure of RRM1-D169G mutant is similar to that of wild-type RRM1, nevertheless, a small local difference was observed in the β turn (Turn6) containing the D169G mutation. Superposition of the two proteins yielded an average root-mean-square deviation (RMSD) of 0.39 Å for 71 Cα atoms. Both wild-type RRM1 and D169G mutant exhibit similar affinity for single-stranded (TG)_15_ DNA (K_d_ of 20.6 ± 1.8 nM vs. 14.2 ± 1.7 nM)^9^ and protein-DNA interface interactions (data not shown). However in the D169G mutant, the β turn (Turn6) connecting the β4 and β5 strands, which contains the D169G mutation, shifted slightly due to the loss of the hydrogen bond between D169 and T115 (Fig. 5D,E). The largest shift in the β turn is located around the mutation site G169 (chain A) whose Cα atom was shifted by 0.8 Å as compared to that of D169 of wild-type RRM1 (chain A) (see Fig. 5F). The β turn in all the four RRM1-D169G structures in the asymmetric unit has a similar shift of 1.0 Å (G169, Cα atom) on average as compared to wild-type RRM1 (D169, Cα atom), suggesting that this shift is inherent for the D169G mutant. In summary, the crystal structure of the TDP-43 RRM1-D169G domain reveals a local conformational change in the β turn containing the D169G mutation due to the loss of a hydrogen bond as compared to wild-type TDP-43.

### The D169G mutant has increased hydrophobic core interactions

To understand why a local conformational change in Turn6 in the TDP-43 RRM1 is linked to the increased stability, the crystal structures of the RRM1 domain of the wild-type and D169G mutant were subjected to multiple molecular dynamics simulations at a mean temperature of 300 K and pH 7 for a total sampling period of at least 200 ns (see Methods). The simulation protocol adopted maintained the overall protein structure, as evidenced by an average RMSD of 1.0 Å (wild-type) and 0.9 Å (D169G mutant) for the protein backbone atoms from those in the initial structures. To detect subtle conformational changes, the Cα–Cα and SC–SC (where SC denotes the center of mass of the side chain) distances between wild type RRM1 and RRM1-D169G mutant were compared. Furthermore, the pairwise interactions between all residues were decomposed into van der Waals and electrostatic contributions.

**Figure 6:**
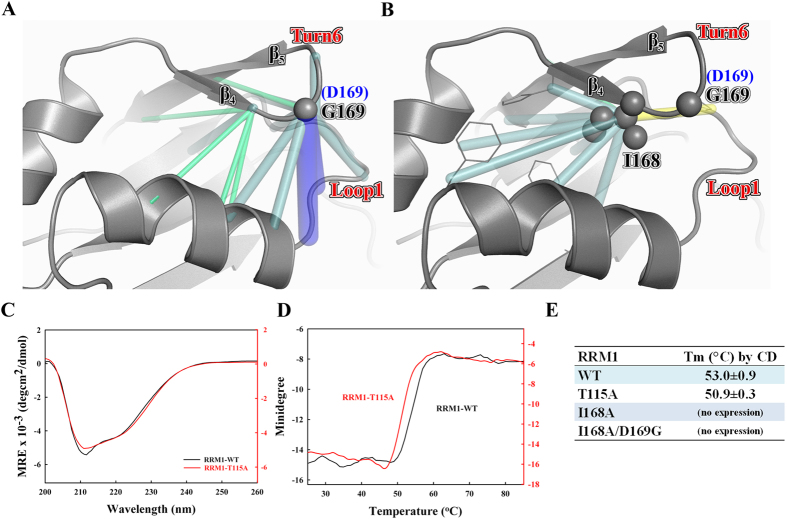
The reinforcement of the hydrophobic interactions in the RRM1 with the D169G mutation as revealed by molecular dynamics simulations. Compared to the wild-type RRM1 simulations, the backbone–backbone distances (**A**) and side chain–side chain distances (**B**) that are shorter in the D169G MD simulations are connected by blue, cyan and green lines, which indicate reductions in the 2–1, 1–0.5 and 0.5–0.3 Å, whereas those that are longer (by 0.5–1 Å) in the D169G MD simulations are connected by yellow line. The carbon atoms of residues G169 and I168 are shown as spheres. (**C**) The CD spectra of wild-type RRM1 and RRM1-T115A mutant. (**D**) The thermal melting points of wild-type RRM1 and RRM1-T115A mutant were measured by CD at a wavelength of 218 nm. (**E**) The apparent thermal melting points of wild-type and mutated RRM1.

The simulation analyses suggest that the D169G mutation results in enhanced packing (van der Waals) interactions between Ile168 and several residues involved in the hydrophobic folding of the domain: The D169G mutation enabled the Turn6 backbone to move closer to Loop1: compared to wild-type D169, the Cα–Cα distance between G169 and the residues located on Loop1 and on the N-terminal part of Helix 1 was reduced by roughly 1 Å on average as compared to D169 (Fig. 6A). This in turn pushed the neighboring Ile168 side chain toward the inner hydrophobic core of the domain. Consequently, Ile168 mediated more packing interactions with the surrounding hydrophobic residues (Fig. 6B): compared with the wild-type protein, the van der Waals energy of Ile 168 in the D169G mutant is more favorable by 1.5 kcal/mol, while that of His166, Cys173, or Phe124 is stronger by 0.25–0.40 kcal/mol. The increased hydrophobic interactions in the inner core of the domain offer a plausible explanation as to why the D169G mutant is more stable than the wild-type protein.

To further confirm that Ile168 is a key residue in the inner hydrophobic core of RRM1 domain, we mutated Ile168 into alanine and generated two mutants, RRM1-I168A and RRM1-I168A/D169G. However, these two mutants could not be expressed, indicating that Ile168 is indeed crucial for protein folding. To assess the importance of the hydrogen-bonding interactions between D169G and Thr115 for protein stability, we also mutated Thr115 to alanine in RRM1. Thr115 is located in Loop1 whereas D169G is located in Turn6 in RRM1. The RRM1-T115A mutant could be purified and its secondary structure was similar to that of wild-type RRM1 as revealed by CD (Fig. 6C). However, RRM1-T115A exhibited lower thermal stability with a lower melting point of 50.9 °C compared to 53.0 °C for wild-type RRM1, as monitored by CD at 218 nm (Fig. 6D,E). This result suggests that the hydrogen-bonding interactions between T115 and D169 seem to affect the overall stability of the RRM1 domain.

### Increased TDP-35 fragments of D169G mutant *in vivo*

**Figure 7:**
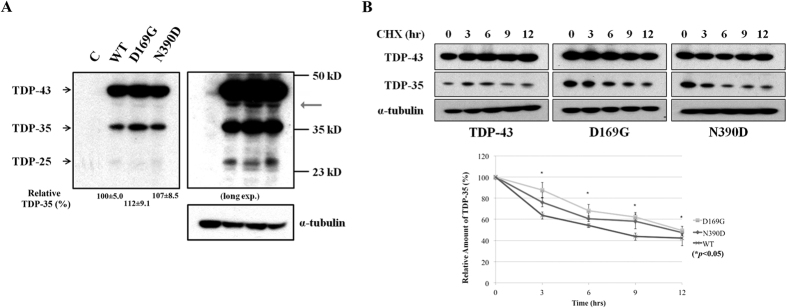
Expression of TDP-43 with D169G mutation in N2A cells produces higher level of TDP-35 fragments with increased stability. (**A**) Stable cell lines of the mouse neuroblastoma (Neuro2a) expressing different TDP-43 proteins were lysed in the RIPA buffer and immunoblotted with anti-His antibodies. Arrows indicated the 43 kD, 35 kD, and 25 kD fragments of TDP-43. Right panel: A 25 kD fragment was observed in the long-exposure blot. Note that the exogenous TDP-43 in stable cells expressing D169G mutant shows higher level of the cleaved TDP-35 (the C-terminal 35 kD fragments) as compared to those of wild-type TDP-43 and N390D mutant. (**B**) The degradation pattern of TDP-35 was assayed by treating cells with cycloheximide and collecting the cells at different time points. The His-tagged TDP-43 proteins were blotted by anti-His antibodies and α-tubulin was blotted by anti-tubulin antibodies in the same membrane. TDP-35 with D169G mutation was degraded slower than those of wild-type TDP-43 and N390D mutant. The relative amounts of TDP-35 at different hours of cycloheximide treatment are plotted in the lower panel.

To further examine the stability and cleavage pattern of TDP-43 *in vivo*, we generated the mouse neuroblastoma (Neuro2a) stable cell lines constitutively expressing the wild-type TDP-43 and the D169G mutant, using the N390D mutant as the positive control. The ALS-associated N390D mutant is more stable and has a higher steady-state level in Neuro2a cells, and it causes higher apoptotic death rate than wild-type TDP-43^58^. To examine the cleavage pattern of TDP-43, the cell extracts of the stable Neuro2a cells expressing wild-type TDP-43, D169G and N390D mutants with a C-terminal His-tag were lysed in RIPA buffer and the protein steady-state levels were analyzed by Western blotting using anti-His-tag antibodies. We found that stable cells expressing D169G mutants had a slightly higher steady-state level of TDP-35 (112 ± 9.1%) compared to that of wild-type TDP-43 (100 ± 5.0%) and N390D mutant (107 ± 0.85%) (Fig. 7A, left panel). The increased level of TDP-35 was clearly observed in the long exposed gel (Fig. 7A, right panel). Interestingly, the stable cells expressing D169G mutant had a lower amount of TDP-25 compared with wild-type TDP-43 and N390D mutant (right panel of Fig. 7A). We noted that extra TDP-43 bands were observed in the long exposed gel (marked by an arrow in Fig. 7A) and the level of these extra bands were slightly increased in the cell expressing TDP-43 D169G mutant. It has been shown previously that TDP-43 can be digested by caspases^50^ and calpain^59^ to produce digested fragments, including TDP-35 and TDP-25. Moreover, similar to D169G digested faster by caspase 3, the ALS-linked mutants, A315T and M337V, were digested faster by calpain-I^59^. We thus suggest that some other proteases other than caspases were likely involved in digesting TDP-43 into the C-terminal fragments, whose production are affected by the D169G mutation and important for TDP-43 clearance. In comparing only TDP-35 and TDP-25 fragments, our *in vivo* result shows that TDP-43 with the D169G mutation was cleaved more efficiently into TDP-35, but not TDP-25. This result is consistent with a recent study^50^ showing that transient expression of TDP-43 D169G mutant produced a higher level of TDP-35 in human U2OS-derived stable cell line, as compared to wild type by pulse-chase analysis.

Several TDP-43 mutants have been shown to exhibit an increased protein half-life in different cell models^43^,^47^,^58^. The full-length D169G and N390D TDP-43 mutants have increased half-lives compared to wild-type TDP-43^44^,^58^. However, it is not known if the D169G mutation stabilizes TDP-35 in the cell expressing the D169G mutant. We thus further treated the stable cells with cycloheximide to inhibit protein synthesis and collected the cells at various time points to monitor the stability of TDP-43 (Fig. 7B). Notably, the level of the cleaved C-terminal fragment TDP-35 was significantly higher in the stable cells expressing D169G mutant compared to wild-type and N390D mutant at each of the time points of cycloheximide treatment (Fig. 7B). Moreover, TDP-35 with the D169G mutation was stabilized and degraded slower than wild-type TDP-35, as shown in the bottom panel of Fig. 7B. In summary, the *in vivo* study also shows that expression of the D169G TDP-43 mutant generates a higher level of accumulated TDP-35 than wild type and TDP-35 with the D169G mutation is more stable than wild-type TDP-35.

## Discussion

In this study we tested six ALS-linked TDP-43 mutants and found one mutant D169G with significantly higher thermal stability than the wild-type protein. The crystal structure and MD simulations further reveal that the higher stability of D169G compared to wild-type TDP-43 is due to a local conformational change in the β turn region containing the D169G mutation and a reinforcement of the hydrophobic interactions in the core of RRM1. Although mutation of D169 to G in TDP-43 has been reported to result in enhanced protein stability^9^ and increased cellular half-life^44^,^50^, the studies herein explain why this mutation increases protein stability and half-life.

Our studies also show that the TDP-43 D169G mutant could be cleaved more efficiently by caspase 3 to generate higher levels of TDP-35 *in vitro*, and the expression of the D169G TDP-43 mutant in mouse neuroblastoma cells consistently generated higher levels of TDP-35 than that of the wild-type TDP-43 (Figs 4 and 7). Why would a more stable TDP-43 D169G mutant be cleaved by caspase 3 more efficiently to generate a higher level of TDP-35, but not TDP-25? Since the stabilization effect resulting from the D169G mutation is more pronounced in smaller protein constructs such as RRM1 (+7.6 °C) than larger constructs such as N-RRM12 (+2.0 °C), the smaller TDP-35 could be stabilized more than the full-length TDP-43 by the D169G mutation and hence, the TDP-43 D169G mutant is more susceptible to caspase 3 digestion. This enhanced stabilization by the D169G mutation does not apply to TDP-25, as it does not contain the RRM1 domain with the D169G mutation. Thus, our results suggest that the enhanced stability in the D169G mutant is linked to the increased proteolytic digestion by caspase 3 as well as to increased levels of full-length TDP-43 and cleaved fragments of TDP-35.

While the D169G mutation in TDP-43 increases protein stability and half-life, cellular expression of D169G mutant is related either to decreased levels of TDP-43 aggregation^44^,^45^ or increased number of aggregates in SH-SY5Y cells^55^. Moreover, expression of TDP-43 D169G mutant is cellular toxic, inducing more severe cell death than wild-type TDP-43^50^. However, the severe cell death phenotype of D169G mutation in TDP-43 could be largely alleviated by the additional introduction of a D89E mutation^50^. The D89E/D169G double mutant is resistant to caspase digestion in the generation of TDP-35^50^, implying that high levels of TDP-35 is likely the major component in inducing cell death. Furthermore, TDP-35 has been suggested to form cytoplasmic inclusions that can further recruit full-length TDP-43 to cytoplasmic deposition and the level of TDP-35 is closely related to the formation of cytoplasmic TDP-43 inclusions^51^,^52^,^60^. All these results indicate that the D169G mutation in TDP-43 leads to an accumulation of TDP-35, which is the pathological component associated with cell death and protein accumulation.

TDP-43 shares similar features with the Aβ precursor protein (APP) in Alzheimer’s disease, as both proteins are cleaved before aggregation. APP is endoproteolytically cleaved by β and γ secretases to generate Aβ peptides that are the primary components of amyloid deposits in the brain^61^. About 24 mutations in APP have been identified that cause early-onset Alzheimer’s disease^62^. These mutations are located either adjacent to or within the sequence that encodes Aβ peptides, presumably affecting the efficiency of β-secretase cleavage and producing excess Aβ peptides^62^. Similarly, TDP-43 is endoproteolytically cleaved by caspases^48^,^49^ to generate TDP-25 and TDP-35 fragments that are the major components in the ALS and FTLD ubiquitylated inclusions^2^,^3^. Here, we show that mutations in TDP-43 may increase protein stability and caspase 3 cleavage efficiency leading to enhanced levels of the pathogenic TDP-35. Intriguingly, the increased protein stability of TDP-43 D169G mutant only affects cleavage by caspase 3 but not caspases 4, 6 and 7; therefore only the level of TDP-35 is increased but not that of TDP-25. TDP-25 is suggested to be the major intermediate for TDP-43 clearance because the TDP-43 mutants that are more resistant to caspase cleavage generate less TDP-25, resulting in reduced TDP-43 clearance rates and cell death^49^,^50^. However, the role of TDP-35 in protein accumulation and aggregation is less clear. Our findings suggest that the D169G mutation in TDP-43 results in more caspase 3-cleaved TDP-35, which might be a critical component for protein accumulation and cellular toxicity. The C-terminal fragments of TDP-43, TDP-35 and TDP-25 likely play different roles in protein accumulation and clearance. Our results also explain why the increased stability of RRM1 due to the D169G mutation is linked to the accumulation of TDP-43 and TDP-35. They suggest that modulating TDP-43 stability and caspase cleavage efficiency might offer a strategy in the prevention or treatment of TDP-43 linked neurodegenerative diseases.

## Methods

### Construct cloning, protein expression and purification

The cDNA of RRM1 (residues 101–191), N-RRM1 (1–191), N-RRM12 (1–265) and N-RRM12-G (1–330) were amplified from the human *TARDBP* gene and inserted into the BamHI/HindIII sites in the pQE30 vector (Qiagen) for the expression of the N-terminal His-tagged recombinant proteins. The constructs for the single-point mutants of A90V, D169G, G287S, G294A, G298S and A315T and other mutations for structural study (T115A, I168A and I168A/D169G) were generated by QuikChange^®^ Site-Directed Mutagenesis Kit (Agilent Stratagene). All of the plasmids were transformed into *Escherichia coli* M15 strain for overexpression. Small cell culture of ~25 ml was incubated overnight and then added into 1 liter of LB medium with 50 μg ampicillin for cell growth for about 3–4 hours. The cell culture was cooled down to 18 °C and then induced by adding 0.8 mM isopropyl 1-thio-β-D-galactopyranoside (IPTG) and 50 μg ampicillin for 20 hours at 18 °C. The cells were suspended in 50 mM phosphate buffer (pH7.5), 100 mM NaCl and 10 mM β-mercaptoethanol (βME) and lysed by microfluidizer (Microfludics, M-110P) and centrifuged at 12,000 rpm for 30 minutes. The supernatant of cell extracts were applied onto an equilibrated nickel-nitrilotriacetic acid (Ni-NTA) affinity column (Qiagen) with 50 mM phosphate buffer (pH7.5), 100 mM NaCl and 10 mM βME. The N-terminal His-tagged proteins were eluted by a gradient buffer containing 50 mM sodium phosphate buffer (pH 7.5), 100 mM NaCl, 0–1.0 M imidazole and 10 mM βME. The eluted proteins were then purified by the HiTrap heparin column (GE Healthcare) and eluted by a gradient buffer containing 50 mM phosphate buffer (pH 7.5), 0.1–1.0 M NaCl and 10 mM βME. The purified proteins (or mutants) were dialyzed against the buffer containing 50 mM phosphate buffer (pH 7.5), 100 mM NaCl and 10 mM βME for further biochemical assays, or the buffer containing 50 mM HEPES buffer (pH7.5), 100 mM NaCl and 10 mM βME for crystallization and caspase digestion assays.

### Circular dichroism (CD) spectroscopy

All far-UV CD spectra were recorded on the Aviv Circular Dichroism Spectrometer Model 400. Protein samples were diluted with 50 mM sodium phosphate buffer (pH 7.5), 100 mM NaCl and 10 mM βME to give the appropriate protein concentrations (~20 μM) and a total volume of 300 μl for the measurement in a quartz cell with 1-mm path length. The CD spectra were scanned ranging from 260 to 200 nm three times at 25  °C. The final spectra were represented by mean residue ellipticity [θ] (MRE) in deg . cm^2^ . dmol^−1^. Thermal denaturation measurements were performed by monitoring the change in CD signals at wavelength 218 nm from 20 °C to 85 °C in 1 degree intervals with 0.5 minute equilibration time.

### Differential scanning fluorimetry

The differential scanning fluorimetry experiments were carried out by the LightCycler^®^ 480 system (Roche) in triplicate using the protocols described previously^63^. A total of 5 μg of protein sample in 24 μl buffer (50 mM NaHPO_4_ buffer, pH7.5, 100 mM NaCl and 10 mM βME) was incubated at room temperature for 10 minutes. The 125X SYPRO Orange (Invitrogen) dye (1 μl) was then added to the protein and loaded on a multiwell plate (MultiWell Plate 96 White (Roche)) and sealed with LightCycler^®^ 480 Sealing Foil (Roche). The temperature was increased from 20 to 85 °C at a heating rate of 0.012 °C/second and the emitted fluorescence at 568 nm (excited at 483 nm) was recorded every 0.02 °C. The melting temperature (Tm) was calculated by the LightCycler^®^ Protein Melting Analysis.

### *In vitro* caspase digestion assays

The TDP-43 (N-RRM12) (5 μg) and its mutants were incubated respectively with human active recombinant caspase 3 (BD Phamingen), caspase 4 (BioVision), caspase 6 (BioVision), and caspase 7 (BioVision) in a buffer containing 50 mM HEPES (pH 7.5), 100 mM NaCl, 10 mM DTT, 1 mM EDTA, 10% glycerol and 0.1% CHAPS at 37 °C for 2 hours (caspase 4, 6, 7 for 16 hours). The reactions were stopped by heating the mixture at 95 °C for 5 minutes, and the digested protein samples were resolved in 15% Tricine-SDS-PAGE with Coomassie blue staining. The data obtained from three independent experiments were shown as the mean standard deviation (±S.D.). The differences between the variants were appraised by the ANOVA test. Statistical significance P values were determined by an unpaired two-tailed Student’s *t*-test which associated comparisons between the wild-type and mutant proteins.

### Crystallization, structure determination and refinement

The purified human RRM1 and RRM1-D169G were mixed with the single-stranded DNA with a sequence of 5′-GTTGAGCGTT-3′ in a molar ratio of 1:1.2. The crystals of RRM1-DNA complex were grown by hanging drop vapor-diffusion method at room temperature by mixing 1 μl of protein-DNA solution with 1 μl of reservoir solution containing 0.12 ammonium acetate, 20% PEG 3350 and 0.08 BIS-TRIS pH 5.5. The crystals of RRM1-D169G/DNA complex were grown in the reservoir solution containing 0.05 M CH_3_COONH_4_, pH 5.0 and 15% v/v Jeffamine ED-2001, pH 7.0.

The X-ray diffraction data of hRRM1/DNA were collected by Rigaku FR-E + SuperBright X-ray generator with Saturn 944 + CCD detector. The diffraction data of RRM1-D169G/DNA were collected at SPXF beamline BL15A1 at NSRRC (Taiwan). The diffraction data were processed and scaled by HKL2000. The RRM1/DNA complex was crystallized in the hexagonal space group P6_5_22 with two complexes per asymmetric unit, whereas the RRM1-D169G/DNA complex was crystallized in the trigonal space group P3_2_21 with four complexes per asymmetric unit. The two complex structures were solved by molecular replacement using the wild-type RRM1 structure (PDB entry code: 4IUF) as the search model. The crystal structures were refined by PHENIX and the data collection and refinement statistics are summarized in Table 2. The structure factors and coordinates were deposited in RCSB database with an accession code of 4Y0F for RRM1/DNA complex and 4Y00 for RRM1-D169G/DNA complex.

### Molecular dynamics (MD) simulation

The crystal structure of the wild-type RRM1 reported previously (PDB entry code: 4IUF) and RRM1-D169G determined in this study (PDB entry code: 4Y00) were subjected to molecular dynamics simulation. All simulations were performed using the OpenMM5.2 CUDA version of CHARMM version 37, and the CHARMM36 all-atom parameter set. The systems were first submitted to a 30-ps heating phase (from 0 to 300 K), followed by a 500-ps equilibration and a 20-ns production phase in the NPT ensemble. Simulation analysis was performed at room temperature (300 K) with an integration step set to 2 fs, except for the heating phase for which a 1-fs time step was used. Constant pressure was ensured by the use of a Langevin piston with a friction coefficient of 10 ps-1 at a reference pressure of 1 bar. Coordinates were saved every picosecond. All non-bonded interactions were truncated at 16 Å, van der Waals interactions were switched at 12 Å and electrostatic interactions were treated via the particle mesh Ewald summation method. To increase the sampling, several replicates of 20 ns each were performed, rather than a single long-trajectory. As four copies of RRM1-D169G were present in the crystal structure, 3 replicates of each copy were simulated. As for the wild-type RRM1, only one copy was found in the crystal structure, 10 replicates were performed. Therefore, in total, at least 200 ns of sampling was obtained for each form.

### Cell cultures, stable cell generation and protein degradation assays

The cDNA of the wild-type TDP-43, D169G and N390D mutants were cloned into the XhoI/ SacII sites of a pcDNA3.1-Myc/His (B) vector (Invitrogen) with a C-terminal Myc/ His tag. Mouse neuroblastoma (Neuro2a) cells were cultured in Modified Eagle Medium with 10% heat-inactivated fetal bovine serum (Invitrogen), 1% penicillin-streptomycin (Invitrogen) and 1% sodium pyrubate (Invitrogen). For generating stable cell lines, cells were subcultured and transfected in 6-well plates with TDP-43 constructs by selection in 500 mg/ml G418 (Promega) for two weeks and limiting dilution was used to generate monoclonal cell lines.

For detecting the TDP-35 protein degradation, stable cells expressing wild-type TDP-43 and mutant (D169G and N390D) constructs were treated with cycloheximide (100 μg/ mL; Sigma). At various time points thereafter, the transfected cells were lysed and the amounts of the TDP-43 proteins were measured by Western blot analysis using the anti-His antibody (Proteintech). Cells were lysed in RIPA buffer (0.1% SDS, 1% Nonidet P-40, 0.5% sodium deoxycholate, 5 mM EDTA, 150 mM NaCl, 50 mM Tris-HCl, pH 8.0) supplemented with protease inhibitors (Roche) and phosphatase inhibitors (Sigma). The protein concentrations of the lysates were measured using the Bio-Rad protein assay reagent on a Beckman Coulter DU-800 machine. The lysates were then resolved by 12% SDS–PAGE and immunoblotted with the anti-His antibodies. Quantification of the immunoblot band intensities was performed with use of the Image J software (NIH) as described previously^58^.

## Additional Information

**How to cite this article**: Chiang, C.-H. *et al*. Structural analysis of disease-related TDP-43 D169G mutation: linking enhanced stability and caspase cleavage efficiency to protein accumulation. *Sci. Rep.* **6**, 21581; doi: 10.1038/srep21581 (2016).

## Supplementary Material

Supplementary Information

## Figures and Tables

**Table 1:** Thermal melting points (Tm) of TDP-43 measured by circular dichroism (CD) and differential scanning fluorimetry (DSF).

**TDP-43**		**Tm (°C) byCD**	**Difference (°C)**	**Tm (°C) by DSF**	**Difference (°C)**
N-RRM12-G	WT	39.3 ± 0.8		39.5 ± 0.2	
	A90V	37.7 ± 0.5	−1.6		
	D169G	42.3 ± 0.8	+ 3.0	41.9 ± 0.1	+ 2.4
	G287S	38.5 ± 0.7	−0.8		
	G294A	39.8 ± 0.2	+ 0.5		
	G298S	38.9 ± 1.6	−0.4	40.4 ± 0.4	+ 0.9
	A315T	38.8 ± 0.4	−0.5	40.1 ± 0.1	+ 0.6
N-RRM12	WT	43.6 ± 0.9		41.4 ± 0.4	
	D169G	45.6 ± 0.5	+2.0	43.6 ± 0.6	+ 2.2
N-RRM1	WT	38.7 ± 0.3		47.6 ± 0.5	
	D169G	45.5 ± 0.5	+6.8	51.5 ± 0.9	+ 3.9
RRM1	WT	53.0 ± 0.9		55.3 ± 0.1	
	D169G	60.6 ± 0.5	+7.6	59.7 ± 0.3	+ 4.4

**Table 2:** Crystallographic statistics of RRM1/DNA and RRM1-D169G/DNA complex.

	**RRM1/DNA**	**RRM1-D169G/DNA**
Data collection and processing		
Wavelength (Å)	1.5418	1.0000
Space group	P6_5_22	P3_2_21
Cell dimensions (Å)	a = b = 100.03,	a = b = 97.64
	c = 98.14	c = 96.87
Resolution (Å)	2.65 (2.74–2.65)	3.00 (3.13–3.00)
Observed reflections	81,006	74,020
Unique reflections	8,322	11,889
Redundancy^a^	9.7 (4.6)	6.2 (6.6)
Completeness^a^ (%)	89.9 (82.77)	97.2 (100)
Rsym^a^	4.3 (39.0)	6.2 (51.8)
*I*/σ (*I*)^a^	44.43 (5.14)	18.1 (3.72)
Refinement statistics		
Resolution range	27.70–2.65	16.85–3.0
Reflections (work/test)	7,194/784	9,600/1073
R-factor/ R-free (%)	24.04/28.83	26.58/29.49
Number of atoms		
Protein	2,527	5,034
Solvent molecules	37	24
Model quality		
r. m. s. deviations in		
Bond length (Å)	0.002	0.003
Bond angle (^o^ )	0.66	0.62
Average B-factor (Å^2^)	44.0	83.0
Ramachandran plot (%)		
Most favored	98.70	94.70
Additionally allowed	1.30	5.30
Generously allowed	0	0
Disallowed	0	0

^a^The last shell statistics are listed in parenthesis.
