# Supplementary material for: Structural analysis of disease-related TDP-43 D169G mutation: linking enhanced stability and caspase cleavage efficiency to protein accumulation
Source: Sci Rep. 2016 Feb 17;6:21581. doi: 10.1038/srep21581 (PMC4756693; doi:10.1038/srep21581)
Supplement: Supplementary Information [file srep21581-s1.pdf]

Supplementary Information

Structural analysis of disease-related TDP-43 D169G mutation: linking enhanced stability and caspase cleavage efficiency to protein accumulation

Chien-Hao Chiang[1,2], Cédric Grauffel[3], Lien-Szu Wu[1], Pan-Hsien Kuo[1], Lyudmila G. Doudeva[1&], Carmay Lim[3], Che-Kun James Shen[1] and Hanna S. Yuan[1*]

Supplementary data: Supplementary Figure 1

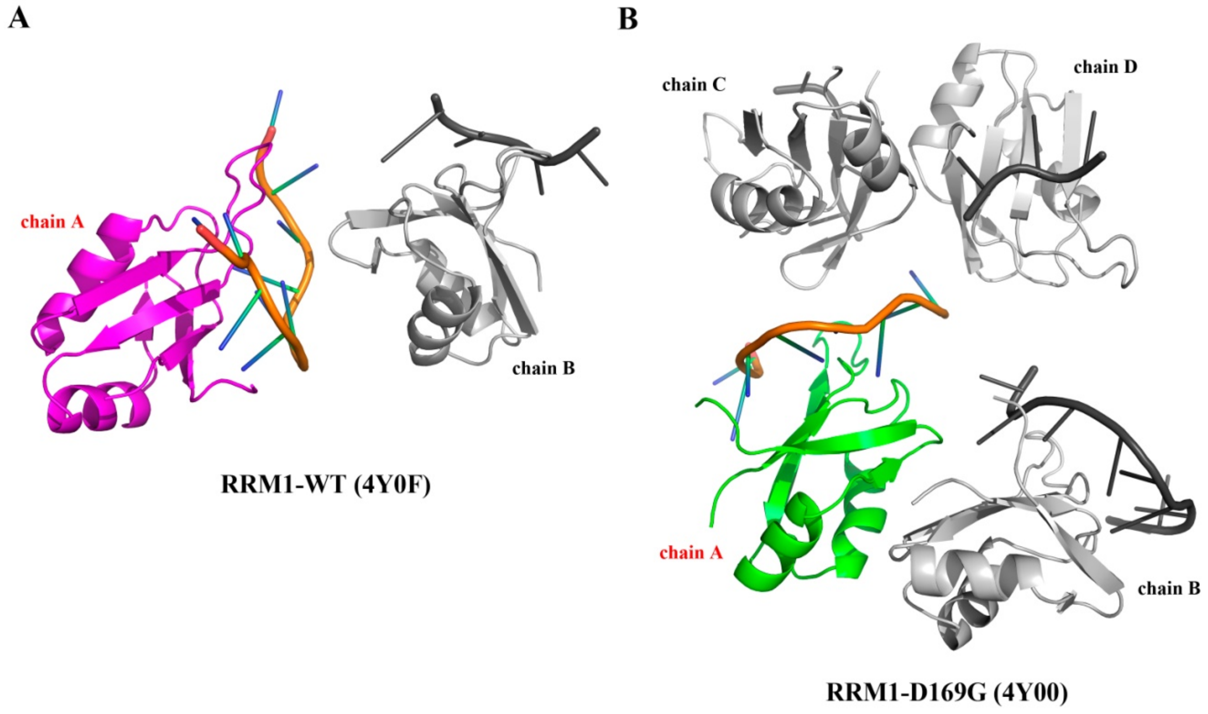

Supplementary Figure 1. The overall crystal structures of RRM1-WT and RRM1-D169G. (A) The final refined model of RRM1/DNA complex contains two RRM1 polypeptide chains (chain A and B) and two single-stranded DNA per asymmetric unit. The PDB accession code for RRM1/DNA complex is 4Y0F. (B) The final refined model of RRM1-D169G/DNA complex contains four RRM1 polypeptide chains (chain A to D) and four single-stranded DNA per asymmetric unit. The PDB accession code for RRM1-D169G/DNA complex is 4Y00. For clarity, only a single RRM1/DNA (chain A in magenta) and RRM1-D169G/DNA (chain A in green) were used for structural presentation and comparison.
